# MEDICAID-MANAGED LTC: A NATIONAL OVERVIEW

**DOI:** 10.1093/geroni/igz038.847

**Published:** 2019-11-08

**Authors:** Larry Polivka

**Affiliations:** Florida State University, Tallahassee, Florida, United States

## Abstract

Most of the states now have Medicaid LTC programs administered by corporate HMOs. Several states, however, still have programs administered by non-profit community based organizations, most of which are members of long standing Aging Networks which grew out of the Older Americans Act in the 1970s. This paper will offer a comparative overview of these models of LTC administration including a typology designed to identify major cultural and political differences between the states with and without corporate managed LTC models and an analysis of the available information regarding their comparative costs and outcomes, mainly access to care and quality of care. The paper will conclude with an assessment of the implications of the information presented for the future of Medicaid LTC policy and politics at the state and federal levels and for the future of LTC advocacy and accountability across the states.

